# Ameliorating Effects of Iron and Zinc on *Vigna mungo* L. Treated with Tannery Effluent

**DOI:** 10.1155/2014/910497

**Published:** 2014-11-19

**Authors:** Shefali Srivastava, Kumkum Mishra, Pramod Kumar Tandon

**Affiliations:** Department of Botany, University of Lucknow, Lucknow 226 007, India

## Abstract

Different dilutions, that is, 25, 50, 75, and 100%, of tannery effluent (TE) were chosen for the present study to assess the phytotoxic effects on *Vigna mungo* L. For amelioration purposes, different levels and combinations of iron and zinc were supplied to the plants along with 50% TE that is chosen on the basis of prior test under Petri dish culture. Cytotoxic and biochemical analysis and plant tolerance index (PTI) of plant were observed. Mitotic index deceased with increase in effluent concentration whereas abnormality % was increased. The pigments (chlorophyll a, total, and carotenoids) were decreased with increasing treatment levels of TE at both growth stages. However, carotenoid content increased significantly at all dilution levels of TE after first growth stage. Chlorophyll b was increased significantly after 35 days of growth but decreased after 70 days. The protein contents were also significantly decreased with increase in all TE treatments and increased significantly in zinc recovery treatments. Activities of catalase and peroxidase enzymes were significantly affected and increased significantly with effluent treatments. PTI showed an enhanced tolerance capacity of plant with treatment of iron and zinc. A negative correlation was found (*r* = −0.97) between plant height and different dilutions of effluent whereas it was positively correlated (*r* = 0.95) with iron and zinc treatments. The study represents the ameliorative effect of iron and zinc for phytotoxic damage in *V. mungo* caused by tannery effluent.

## 1. Introduction

With the increasing demand for leather and leather products, both for indigenous use as well as for export, a large number of vegetable and chrome tanneries have mushroomed in India, especially in states like Tamil Nadu, Uttar Pradesh, and West Bengal. There are around 3000 tanneries in India with an annual processing capacity of 70,000 tones of hides and skins [1]. To process the leather, animal hide and skin are treated with various chemicals, in which chromium sulphate is mainly used for chrome tanning in tanneries [2]. An indiscriminate use of chromate ensures the presence of chromium in effluent coming out from the leather industry [3]. Presence of chromium in surplus makes it unfit to be used for irrigation of crop field [4].

On humans and animals, chromium can have carcinogenic and genotoxic effects and may have immunotoxic effects also [5]. However, in plants, chromium interferes with uptake, translocation, and accumulation of calcium, potassium, magnesium, phosphorus, boron, and copper by plant tops and aggravates iron deficiency and chlorosis by interfering with iron metabolism. Plants show different responses with effluents at different stages of development and also the magnitude of toxicity depends upon the plant species exposed.

Chromium toxicity leads to reduced photosynthesis and hampered chloroplast synthesis [6]. High chromium concentration in plants growing near by the discharging unit showed leaf necrosis with high foliar Cr accumulation in vegetables and fruit trees [7]. It is known to tempt the production of reactive oxygen species and induces oxidation of lipids. High Cr concentration in irrigating water decreases the uptake of many essential nutrients like iron, zinc, copper, and so forth [8] in plants. Also, use of this waste water for irrigation purposes poses a serious threat to plants due to excessive metal accumulation in the plant parts and their effects have been extensively studied worldwide [9–11]. In recent years, researches have been focused to find out the new technologies for waste water treatment and heavy metal recovery from them. However, very little information is available about the amelioration efforts for effluent toxicity before its discharge to rivers or streams. In the view of the above, this study aims to study the physiological and cytogenetic effects of tannery effluent on* Vigna mungo *and to find out ameliorating effects of Fe and Zn on phytotoxicity induced in plants with effluent treatment.

## 2. Materials and Methods

The seeds of urad (*Vigna mungo *L.) were sterilized in 0.1% HgCl_2_ solution to remove any surface contamination, washed with redistilled water for three changes, and then soaked in water overnight. The soaked and uncontaminated seeds were evenly sown in earthen pots containing soil mixed with cow dung in 3 : 1 ratio under glasshouse conditions. Ten seeds were sown in each pot and five were maintained later. Four different dilutions of tannery effluent (TE) were prepared using tap water. For recovery of plant damage, levels 5, 10, and 15 ppm of zinc sulphate (ZnSO_4_) and iron sulphate (FeSO_4_) were also prepared and added in 50% effluent treatment. Different combinations of zinc and iron, that is, 5 ppm Zn + 5 ppm Fe, 10 ppm Zn + 10 ppm Fe, and 15 ppm Zn + 15 ppm Fe, along with 50% effluent concentration were also given to plants. The final treatments were T1 = Control (distilled water), T2 = 25% TE, T3 = 50% TE, T4 = 75% TE, T5 = 100% TE, T6 = 50% TE + 5 ppm Zn, T7 = 50% TE + 10 ppm Zn, T8 = 50% TE + 15 ppm Zn, T9 = 50% TE + 5 ppm Fe, T10 = 50% TE + 10 ppm Fe, T11 = 50% TE + 15 ppm Fe, T12 = 50% TE + 5 ppm Zn + 5 ppm Fe, T13 = 50% TE + 10 ppm Zn + 10 ppm Fe, and T14 = 50% TE + 15 ppm Zn + 15 ppm Fe. Analysis was done in 35- and 70-day-old plants.

Physicochemical analysis of effluent was carried out following standard methods by APHA [12]. Pigments' estimation was done by using plant extract in 80% acetone by the method of Arnon [13] using spectrophotometer (Chemito UV 2000) at the wavelength of 645 nm, 663 nm, 510 nm, and 470 nm for estimation of total chlorophyll, chlorophyll a, chlorophyll b, total, and carotenoid, respectively. Chlorophyll and carotenoid content was calculated in mg g^−1^ fw by the method of [14]. Total protein contentswere estimated by using the method of Lowry et al. [15] using egg albumin as a reference. Catalase activity was determined following the modified method of Bisht [16]; using 10% extract of seedling was used for the estimation of catalase activity in terms of mL H_2_O_2_ split/gm fresh weight of tissue. Peroxidase activity was measured by using the modified method of Luck [17]; using 2.5% extract was used for the estimation of peroxidase activity in the terms of units/gm fresh weight of tissue. For cytological studies, root tips were fixed in Carnoy's fluid as per method of Darlington and La Cour [18] and slides were prepared to assess the mitotic index and abnormality percent. Measurements for height, fresh weight, and dry weight were taken after harvesting of plants. Plants tolerance indices were calculated according to the method described by Taylor and Foy [19] with modifications. All the experiments were carried out in triplicate and the data were subjected to standard deviation and analysis of variance by one way method.

## 3. Results

### 3.1. Physicochemical Characteristics of Effluent

Tannery effluent was with alkaline pH and no dissolved oxygen (DO) (Table 1). It contained high amounts of suspended solids with high BOD and COD. Besides, effluent contained 31.60 mg L^−1^ chromium, 1.80 mg L^−1^ zinc, and 18.90 mg L^−1^ iron concentrations. Presence of high suspended solids creates hindrance in light penetration that result in amount of solids that affects the light penetration into the soil resulting in poor growth of microorganisms in soils, reduction in photosynthesis, and respiration and ultimately reduces the soil/water fertility.

### 3.2. Cytotoxic Analysis of Plants

Mitotic index (MI) and abnormality percent in root tips of urad plants (Table 2) were found to be altered with respect to control. The MI decreased and abnormality (%) increased with an increase in effluent concentration. It was observed that 100% effluent concentration could induce 100 abnormality percent.

### 3.3. Metabolic Analysis of Plants

**Figure 1:**
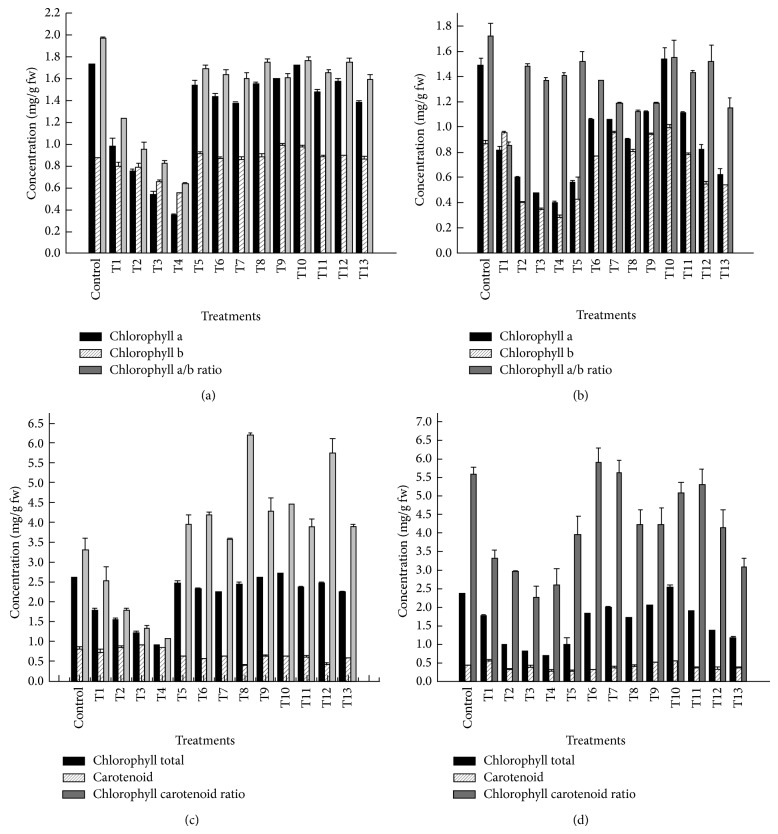
Effect of tannery effluent, Fe, and Zn on chlorophyll and carotenoid concentration of* V. mungo* after 35 and 70 days of growth.

The photosynthetic pigments were decreased with increase in effluent concentration (Figure 1; Table 3) after 35 and 70 days of growth. Chlorophyll a content in leaves was found to be decreased significantly over control at both growth stages. However, chlorophyll b showed a different trend. It decreased at all dilution levels of TE but increased at all recovery treatments being highest under 50% TE + 15 ppm Fe with 111 and 114% increase over control after 35 and 70 days of growth. Total chlorophyll concentration decreased significantly after both growth stages though a significant increase of 103% was recorded at 50% TE + 15 ppm Fe after 35 days of growth. The chlorophyll a/b ratio was found to be decreased significantly after 35 and 70 days of treatment of TE. With increase in effluent dilution, there was a gradual increase in carotenoid content with maximum 113% at 75% TE whereas under recovery treatments it showed a reverse trend. After 70 days of growth, it decreased significantly at 100% TE, 50% TE + 5 ppm Zn, and 50% TE + 10 ppm Zn whereas a significant increase of 127 and 126% was recorded at 25% TE and 50% TE + 15 ppm Fe, respectively, over control. Chlorophyll/carotenoid ratio was decreased significantly at all dilutions of TE, after 35 days of growth, while it increased under recovery treatments with maximum 189% at 50% TE + 5 ppm Fe. The ratio was found maximum under control plants after 70 days of growth.

**Figure 2:**
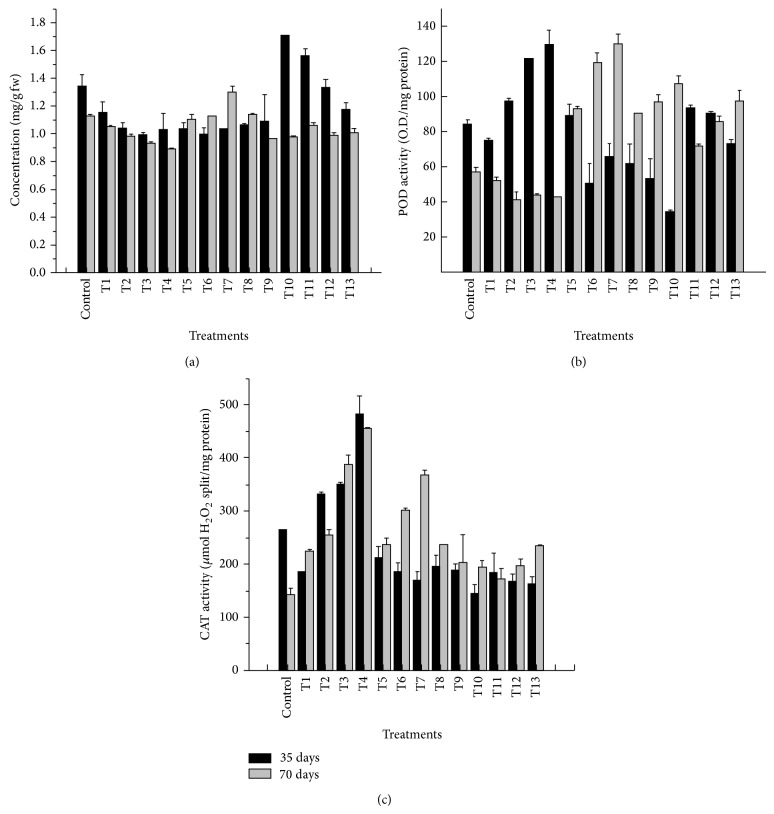
Accumulation of (a) protein and altered (b) peroxidase and (c) catalase activity in* V. mungo* under TE stress.

As compared to control, protein content decreased significantly at all levels of TE except 50% TE + 15 ppm Fe and 50% TE + 5 ppm Zn + 5 ppm Fe where an increase of 127 and 117% was observed (Figure 2(a); Table 3). After 70 days of treatment, protein content was found to be decreased significantly at all dilution levels of TE except at 25% TE. In recovery treatments, it was found to be increased significantly with maximum 116% in 50% TE + 15 ppm Zn over control. Trend of peroxidase activity in plants is given in Figure 2(b) and Table 3. Reduced peroxidase activity was observed at all levels of TE except at 75% TE, 100% TE, and 50% TE + 5 ppm Zn + 5 ppm Fe, where a significant increase was found after 35 days of treatment with lowest activity under 50% TE + 15 ppm Fe. After 70 days of treatment, POD activity was found to be decreased significantly at all dilution levels of TE but was found to be increased significantly at all recovery treatments over control with 227% higher under 50% TE+ 15 ppm Zn. Activity of catalase (Figure 2(c); Table 3) was increased at all dilution levels of TE; however a reverse trend was recorded in recovery treatments, after 35 days of growth. At second growth stage its activity was found to be increased significantly at all levels with 319% increase under 100% TE over control.

**Figure 3:**
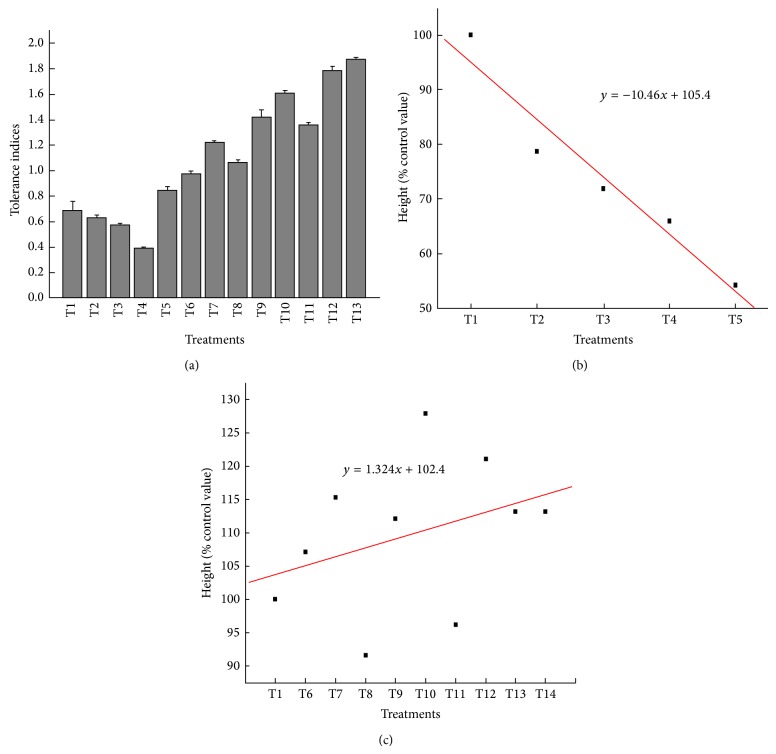
(a) Tolerance indices and percent (b) inhibition and (c) promotion of height of* V. mungo* under TE treatment with recovery dosesof zinc and iron.

Tolerance indices were found to be affected with increase in effluent concentration with increasing trend in all recovery treatments (Figure 3(a)). Maximum plant tolerance index was found in 50% TE + 15 ppm Zn + 15 ppm Fe (187%) but minimum in 100% effluent dilution (39%). A negative correlation (*r* = −0.97) was observed for plant height and effluent dilutions whereas a positive correlation (*r* = 0.95) was observed with recovery treatments (Figures 3(b) and 3(c)).

## 4. Discussion

MI is an important measure for determining the root growth as it accounts for cell division and imitates its frequency. Decrease in growth might have resulted due to decrease in cell division. This might be correlated to the direct binding of metals present in effluents to nucleic acids or due to the action of the enzymes liberated from lysosomes [20]. Similar findings were observed in sugarcane [21]* Vigna mungo* [22] and in* Allium cepa* [23] with the increasing concentrations of chromium.

Metals are thought to reduce the chlorophyll biosynthesis by reacting with –SH group of *δ*-amino levulinic dehydrates. Thus depleted chlorophyll contents in plants treated with effluent enriched with chromium might be attributed to both altered chlorophyll biosynthesis and replacement of Mg ions in the porphyrin ring [24]. Also this decrease in chlorophyll contents is due to chromium that competes for iron at functional site [24]. Providing iron along with water increases the availability of iron at porphyrin ring. Increase in photosynthetic pigment was assumed to be due to the supplementation of these two essential nutrients as also reported by Vàzquez et al. [6] and Kamlesh et al. [25]. Zinc enhances the chlorophyll concentration by acting as an important structural and catalytic component of proteins and enzymes and as cofactor for normal development of pigment biosynthesis [26]. Carotenoids are supposed to act as free radical-scavengers by electron transfer to their double bond structure and play a significant role in the protection of chlorophyll pigment under stress conditions by quenching the photodynamic reactions, replacing peroxidation, and collapsing of membrane in chloroplasts [27]. A decrease in carotenoid content in* Vigna radiata* was reported by Shefali et al. [28] when irrigated with high concentrations of tannery effluent. The chlorophyll carotenoid ratio is considered as a sensitive indicator of plant stress and photooxidative damage [29]. The degeneration of chlorophyll and carotenoid is the most common response observed in plants exposed to increased accumulation of various heavy metals [30–32].

Zinc and iron are essential components for various enzymes systems for energy production, protein synthesis, and growth regulation. Supply of zinc and iron may enhance the superoxide detoxification mechanism of plants by producing hydrogen peroxide. The metals generate reactive oxygen species in the plants grown under stress conditions which damage photosynthetic apparatus and may also catalyze degradation of proteins through oxidative modification and increased proteolytic activity [33]. Additional supply of zinc facilitates the accumulation of amino acids and increased synthesis of proteins, antioxidative defense, and carbohydrate metabolism [34–36] as also observed in our study. The increase in catalase activity may be due to the increased toxic effect of H_2_O_2_ and ROS produced as a result of membrane damage. Our findings were in accordance with the findings of Baskaran et al. [37] in* Vigna radiate *grown under effluent stress conditions. Decreased activity of catalase in plants under recovery treatments may be due to increase availability of Fe for ferrochelatase catalysed Fe incorporation in protoporphyrin IX. The same trend was also reported by Kamlesh et al. [25] in radish. A decrease in catalase and peroxidase activity was reported in all recovery treatments by Nath et al. [38] in* Phaseolus mungo*. Peroxidase induction was caused by uptake of toxic amount of metals in roots and leaves of various species [39]. Decrease in peroxidase activity was also observed by Shakila and Usha [40] in grains of tannery effluent treated* Vigna radiata. *POD activity was found to increase in plants under recovery treatments, in our study that was found contrary to report of Selvarathi and Ramasubramanian [41] in phytoremediated effluent treated tomato that might have contributed to the increased availability of various essential cations and anions in the effluents [37].

Heavy metal interference in iron metabolism reduces the transport of essential nutrients like potassium and iron to meristematic regions of plants that may also be responsible for reduced plant growth. Shefali and Kumkum [42] reported an increase in fresh weight, dry weight, and chloroplastic pigments, in black gram treated with chromium of which toxicity was ameliorated by the use of iron which was in agreement with our study.

These essential elements (Zn and Fe) might have reduced the toxicity of effluents by making essential nutrient available to plants for their growth. The results, found in the present study, are quite significant and portray a clear picture that though bioremediation is a widely accepted technology for tannery effluents, scope of ameliorating them by providing additional supply of essential nutrients may be worked out for their use in irrigational water for better plant growth and ultimately a safe environment.

## Figures and Tables

**Table 1:** Physicochemical characteristics of tannery effluent.

Parameters	Tannery effluent
Colour	Yellowish brown
pH	8.05
Total dissolved solids	2498
Total suspended solids	920
Dissolved oxygen	NIL
Biological oxygen demand	415
Chemical oxygen demand	165
Chromium (Cr)	31.60
Zinc (Zn)	1.80
Iron (Fe)	18.90

Values are given in mg L^−1^ unless otherwise stated.

**Table 2:** Effect of tannery on mitotic index and abnormality % in *V. mungo* under different dilutions.

Effluent concentration	Mitotic index %	Abnormality %
Control	4.09 ± 0.08	0.29 ± 0.00
25%	3.33 ± 0.16	0.95 ± 0.06
50%	2.38 ± 0.09	2.85 ± 1.12

Mean ± S.E.

**Table 3:** Trend of various parameters over control value in *V. mungo* under TE treatment.

Treatments	Chlorophyll a		Chlorophyll b		Chlorophyll total		Carotenoid		Protein		Catalase		Peroxidase	
	35 days	70 days	35 days	70 days	35 days	70 days	35 days	70 days	35 days	70 days	35 days	70 days	35 days	70 days
Control	1.728	1.493	0.878	0.870	2.606	2.363	0.797	0.423	1.341	1.125	263.33	142.66	84.09	57.12
25% E	−	−	−	+	−	−	−	+	−	−	−	+	−	NS
50% E	−	−	−	−	−	−	+	NS	−	−	+	+	−	−
75% E	−	−	−	−	−	−	+	−	−	−	+	+	+	−
100% E	−	−	−	−	−	−	+	−	−	−	+	+	+	−
50% E + 5 ppm Zn	−	−	+	−	−	−	−	−	−	NS	−	+	−	+
50% E + 10 ppm Zn	−	−	NS	−	−	−	−	NS	−	NS	−	+	−	+
50% E + 15 ppm Zn	−	−	NS	+	−	−	−	NS	−	+	−	+	−	+
50% E + 5 ppm Fe	−	−	NS	−	−	−	−	NS	−	NS	−	+	−	+
50% E + 10 ppm Fe	−	−	+	+	−	NS	−	NS	−	−	−	+	−	+
50% E + 15 ppm Fe	NS	NS	+	+	+	NS	−	+	+	−	−	+	−	+
50% E + 5 ppm Zn + 5 ppm Fe	−	−	NS	−	−	−	−	NS	+	NS	−	+	+	+
50% E + 10 ppm Zn +10 ppm Fe	−	−	NS	−	−	−	−	NS	−	−	−	+	−	+
50% E + 15 ppm Zn + 15 ppm Fe	−	−	NS	−	−	−	−	NS	NS	NS	−	+	−	+

+: increase over control; −: decrease over control; NS: nonsignificant.

## Abbreviations

T1 = 25% E
T2 = 50% E
T3 = 75% E
T4 = 100% E
T5 = 50% E + 5 ppm Zn
T6 = 50% E + 10 ppm Zn
T7 = 50% E + 15 ppm Zn
T8 = 50% E + 5 ppm Fe
T9 = 50% E + 10 ppm Fe
T10 = 50% E + 15 ppm Fe
T11 = 50% E + 5 ppm Zn + 5 ppm Fe
T12 = 50% E + 10 ppm Zn + 10 ppm Fe
T13 = 50% E + 15 ppm Zn + 15 ppm Fe.
